# The Phantom Satiation Hypothesis of Bariatric Surgery

**DOI:** 10.3389/fnins.2021.626085

**Published:** 2021-02-01

**Authors:** Laurent Gautron

**Affiliations:** Department of Internal Medicine, Center for Hypothalamic Research, The University of Texas Southwestern Medical Center, Dallas, TX, United States

**Keywords:** neurology, autonomic nervous system, vagus, gastroenterology, appetite, nociception, vagotomy

## Abstract

The excitation of vagal mechanoreceptors located in the stomach wall directly contributes to satiation. Thus, a loss of gastric innervation would normally be expected to result in abrogated satiation, hyperphagia, and unwanted weight gain. While Roux-en-Y-gastric bypass (RYGB) inevitably results in gastric denervation, paradoxically, bypassed subjects continue to experience satiation. Inspired by the literature in neurology on phantom limbs, I propose a new hypothesis in which damage to the stomach innervation during RYGB, including its vagal supply, leads to large-scale maladaptive changes in viscerosensory nerves and connected brain circuits. As a result, satiation may continue to arise, sometimes at exaggerated levels, even in subjects with a denervated or truncated stomach. The same maladaptive changes may also contribute to dysautonomia, unexplained pain, and new emotional responses to eating. I further revisit the metabolic benefits of bariatric surgery, with an emphasis on RYGB, in the light of this *phantom satiation hypothesis*.

## Introduction

### Satiation in Health and Obesity

A wide range of sensations can be evoked from the gastrointestinal (GI) tract including, but not limited to, pain and warmth (Cervero, 1994; Mulak et al., 2008). However, the sensation that is most frequently experienced in healthy subjects is satiation (Stevenson et al., 2015). Satiation corresponds to the sensation of epigastric fullness (without pain) which accompanies meal termination (Benelam, 2009; Bellisle et al., 2012). In the human literature, the term of satiation also commonly refers to the subjective feeling of satisfaction toward the end of a meal (Benelam, 2009; Bellisle et al., 2012). Because satiation directly leads to meal termination, it is a contributing factor to maintaining a normal feeding behavior (de Graaf et al., 2004). To avoid confusion, I will refrain from using the term of *fullness* because it is inconsistently used to refer either to the feeling of gastric distention or to the persistent lack of hunger between meals, which should correctly be referred to as *satiety* (Bellisle et al., 2012; Andermann and Lowell, 2017). The mechanical deformation of the GI tract is a primary event responsible for satiation. Interestingly, as early as 1911, studies in conscious humans with externalized fistulas established that a feeling of gastric distension can specifically arise from the mechanical deformation of the GI muscularis (but not of its mucosa) (Hertz, 1911; Boring, 1915; Wolf and Wolff, 1943; Nathan, 1981). Likewise, human subjects fed by parenteral means, for which nutrients bypass the GI tract, often complain of not feeling satiation to the same extent as after eating and drinking (Stratton and Elia, 1999). Satiation can be assessed in human subjects during the ingestion of a test meal either by quantifying food intake or by assessing self-reported appetite levels (Benelam, 2009). Admittedly, there are technical difficulties in measuring self-reported levels of satiation in humans including considerable variability between individuals (Bellisle et al., 2012; Gibbons et al., 2019; Nielsen et al., 2019; Gero, 2020). While this article is primarily concerned with human biology, I will consider laboratory animal and human studies in parallel. In laboratory animals, food intake can be measured *post hoc* as an indirect indicator of satiation and appetite levels. For example, the inflation of a gastric balloon in rats significantly reduces spontaneous food intake and leads to early meal termination (Geliebter et al., 1986; Phillips and Powley, 1998).

Satiation can be modulated by many factors including nutritional, sociocultural, genetic, and environmental factors. It is beyond the scope of this article to examine all the physiological factors that influence satiation and additional information on the topic can be found in review articles (Warwick, 1996; Benelam, 2009; Keenan et al., 2015; Hetherington et al., 2018; Kral et al., 2018; Rogers, 2018; Gibbons et al., 2019; Thornhill et al., 2019). However, obesity deserves a special mention. Subjects with a weaker satiation response to fatty foods are predisposed to excessive weight gain and obesity (Blundell et al., 2005) and children who spontaneously eat larger meals tend to gain more weight (Syrad et al., 2016). On the other hand, currently available human data are inherently correlative and complicated by the fact that obtaining accurate measurements of caloric intake and energy expenditure remains challenging. In laboratory animals, many studies have shown that meal size increases in response to a high-fat diet (Farley et al., 2003; Melhorn et al., 2010; la Fleur et al., 2014; Treesukosol and Moran, 2014). The observed increase in meal size tends to occur soon after switching animals to a high-fat diet when they are not yet obese, consistent with the view that diminished satiation may precede excessive weight gain. However, considering the wide range of nutritional and environmental factors that can modulate satiation, the mechanisms responsible for altered satiation in obesity are not known with certainty. One possible mechanism may involve altered gut-brain communication with reduced sensitivity to postprandial cues (Kentish et al., 2012). Another non-exclusive possibility may involve exaggerated hedonic responses to an obesogenic diet (Berthoud, 2012; Licholai et al., 2018).

### Brief Overview of the Neurobiology of Satiation

#### Vagal Afferents

The vagus nerve is a mixed nerve containing both efferent and afferent fibers (Berthoud and Neuhuber, 2019). The latter correspond to the vagal neurons carrying sensory information from the GI tract to the brainstem. Vagal afferents responding to stimuli arising from the GI tract are specifically connected to the medial portion of the nucleus of the solitary tract (Saper, 2002). The stomach itself is innervated by two gastric branches of the subdiaphragmatic vagus nerve that enter the gastric wall at the level of the lower esophageal sphincter before sending smaller offshoots throughout most of the muscularis and mucosa (Wang and Powley, 2007). Within the periphery, afferent endings responding to mechanical events are highly enriched in the muscularis at the levels of the stomach and upper intestines (Ozaki et al., 1999; Fox et al., 2000; Williams et al., 2016). At least two types of specialized vagal terminals known as intramuscular arrays and intraganglionic laminar endings are involved in detecting mechanical events in the stomach (Berthoud et al., 1997; Fox et al., 2000; Powley et al., 2016). In addition to the stomach wall, these specialized vagal mechanoreceptors are present at lower densities in the esophagus and duodenum (Wang and Powley, 2000; Wang et al., 2012). Electrophysiological recordings have established that vagal mechanoreceptors rapidly and linearly respond to the application of varied mechanical stimuli to the stomach wall including stretch and tension (Peles et al., 2003; Kentish et al., 2014). Gastric distension suppresses feeding in a vagally-dependent manner in rats (Phillips and Powley, 1998) and, furthermore, mutant mice lacking vagal mechanoreceptors eat larger meals (Fox et al., 2001; Fox, 2006). Conversely, the selective excitation of vagal mechanoreceptors supplying the muscularis elicits meal termination in genetically engineered mice (Bai et al., 2019). As a remark, the same study found that selective mucosal afferents stimulation does not modify feeding. Hence, there is ample evidence that vagal mechanoreceptors supplying the stomach wall are both required and sufficient for eliciting satiation. At the same time, the sensory integration of postprandial cues at the level of vagal afferents is more complex than often appreciated. In particular, mechanoreceptors activity is modulated by many chemical signals including, most notably, the gut peptide cholecystokinin (CCK) (Gibbs and Smith, 1977; Schwartz et al., 1995; Williams et al., 2016). Thus, the excitability of mechanoreceptors is modulated by numerous factors and researchers are just beginning to understand how GI signals are integrated at the level of vagal endings (Egerod et al., 2019). Finally, communication between the stomach and the brain involves more than just vagal afferents, but also a complex network of enteric and spinal neurons (Furness, 2006; Udit and Gautron, 2013; Sharkey et al., 2018; Spencer et al., 2018). Although subsets of spinal afferents respond to a wide range of noxious and innocuous stimuli in the GI tract (Grundy, 1988; Spencer et al., 2016), their contribution to the postprandial regulation of feeding is not well-known (Berthoud and Neuhuber, 2019).

#### Central Viscerosensory Circuits

**FIGURE 1:**
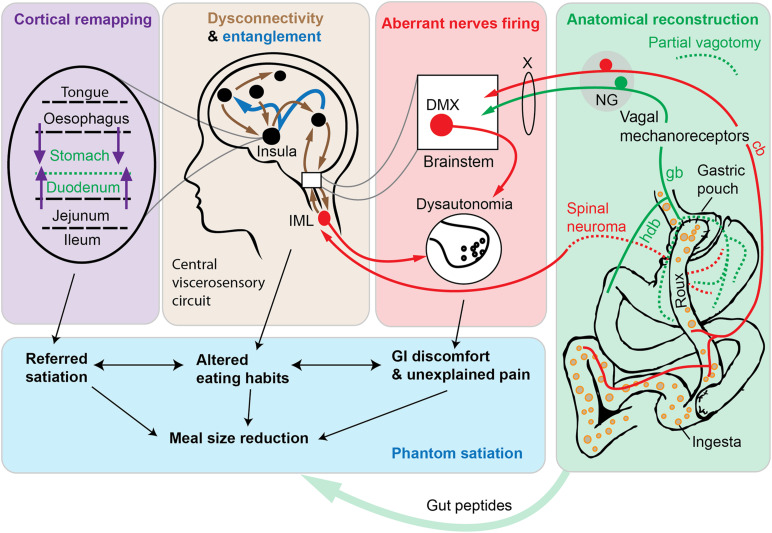
Schematic diagram depicting the phantom satiation hypothesis. According to the observations presented in this article, the metabolic benefits of Roux-en-Y gastric bypass (RYGB) are mediated, at least in part, by a combination of aberrant changes in a complex viscerosensory neural circuit. A key event following RYGB surgery is an unintentional gastric denervation (green box). Available data indicate that RYGB is associated with a gastric denervation (green dotted lines indicate axotomized axons of vagal or spinal origins). The duodenum itself can be conceived as a functionally denervated, in the sense that duodenal afferents cease to be directly stimulated by nutrients and mechanical stimuli. According to our hypothesis, partial vagotomy leads to widespread maladaptive changes in peripheral nerves (red box and arrows) and in central viscerosensory circuits (brown box and arrows). In particular, in animal models of bariatric surgery, sympathetic, parasympathetic, and nociceptive nerves behave as if they were hyperresponsive to a meal (red arrows). At the central level, altered connectivity in many cortical and subcortical areas has been reported after RYGB using brain imaging technologies in human subjects. In addition, the entanglement of viscerosensory modalities (dark blue arrows) may further participate to abnormal emotional and sensory responses to eating. Finally, the remapping of insular cortices involved in gastrointestinal territories representation is likely to occur (purple box and arrows), thereby contributing to viscerosensory anomalies and referred sensations. This neurobiological model, directly inspired by the literature on phantom limbs, predicts that subjects with RYGB may experience exaggerated satiation. Ultimately, reduced meal size and perturbed eating patterns (blue box), among other postprandial anomalies, may contribute to weight loss. Hence, the “phantom satiation” hypothesis reinterprets bariatric surgery as a type of injury—that is to say a procedure that inflicts irreversible anatomical and functional damage—even if an injury with evident long-term health benefits. Abbreviations: cb, celiac branch; deaf., deafferentation; DMX, dorsal motor nucleus of the vagus; gb, gastric branch; hdb, hepato-duodenal branch. NG, nodose ganglion; IML, intermediolateral column; X, vagus nerve.

It is without saying that satiation requires more than the excitation of vagal mechanoreceptors. Vagal afferents are connected, in a multisynaptic manner, to brain networks encompassing integrative cortices and subcortical areas (Min et al., 2011; Hays et al., 2013; Andermann and Lowell, 2017; Cao et al., 2017; Ly et al., 2017). Based on both animal studies and human brain imaging, the regions involved in relaying information of vagal origin to the cortex include, beyond the nucleus of solitary tract, the parabrachial nucleus, and ventrobasal thalamus (Saper, 2000, 2002; Craig, 2002). Without entering into details, the aforementioned brain relays have been linked to the regulation of satiation and meal size in laboratory animals (Dossat et al., 2013; Alhadeff et al., 2014; Cavanaugh et al., 2015; Campos et al., 2016; Zafra et al., 2017). As early as in the 1930’s, studies showed that the electrical stimulation of the vagus nerve altered cortical electrograms (Bailey and Bremer, 1938; O’Brien et al., 1971; Ito and Craig, 2003). Among integrative cortices involved in the processing of gastric distension, vagal information is the insular cortex (Saper, 2002). For instance, based on electrical stimulation experiments in conscious subjects, the stimulation of the human insular cortex causes sensations that are secondary to changes in the GI motility (Pool, 1954). Likewise, many recent brain-imaging studies have described altered activity in the insular cortex in association with the feeling of epigastric distension in humans (Tataranni et al., 1999; Ladabaum et al., 2007; Wang et al., 2008). Importantly, neurons of the insular cortex responding to different visceral territories (stomach, heart, etc.) and sensory modalities (stretch, taste, etc.) are topographically organized (Saper, 2002). There are also insular neurons that can respond to multiple sensory modalities of viscerosensory origin (Saper, 2002). In turn, insular neurons modulate GI functions (Levinthal and Strick, 2020). In summary, one can rightfully conceive the stomach wall as a sensory organ connected to a viscerosensitive neural network specialized in responding to gastric volumetric changes (Figure 1). A detailed knowledge of these brain circuits is not needed to understand the hypothesis described later, and more information can be found elsewhere (Saper, 2002; Mulak et al., 2008; de Araujo et al., 2012; Frank et al., 2016; Andermann and Lowell, 2017).

## Satiation Without a Vagus Nerve

### Experimental and Clinical Vagotomies

Vagotomy is a procedure that consists in denervating vagally-innervated organs to varying extents (Phillips and Powley, 1998). A deafferentation is a procedure consisting of interrupting or destroying the afferent fibers contained in a specific nerve or organ, while minimally interfering with motor fibers (Walls et al., 1995). The regrowth of vagal terminals after vagotomy is possible in rats, but takes weeks and is incomplete (Phillips and Powley, 1998; Powley et al., 2005a). From their inability to feel completely satiated, one would expect vagotomized animals to ingest larger meals and overeat. In laboratory animals, vagotomies can result in increased meal size, hyperphagia, and loss of responsiveness to pharmacological CCK (Smith et al., 1981; Walls et al., 1995; Phillips and Powley, 1998; Schwartz et al., 1999; Sclafani et al., 2003; Powley et al., 2005a). At the same time, vagotomy-induced overeating is often transient, presumably due to compensatory changes at the central level (Walls et al., 1995; Chavez et al., 1997; Phillips and Powley, 1998; Zafra et al., 2003; Reidelberger et al., 2014). Instead, in normal-weight animals, long-term vagotomy and deafferentation have been reported to result in animals eating smaller meals, with some degree of anorexia and weight loss (Mordes et al., 1979; Sclafani and Kramer, 1985; Kraly et al., 1986; Erecius et al., 1996; Leonhardt et al., 2004; Powley et al., 2005a; McDougle et al., 2020). Interestingly, eating smaller meals post-vagotomy is often compensated by more frequent meals, further suggesting significant brain adaptations (Powley et al., 2005a). Likewise, in varied models of rodent obesity, vagotomy, and deafferentation have repeatedly been shown to reduce feeding and body weight gain (Cox and Powley, 1981; Ferrari et al., 2005; Powley et al., 2005a; Stearns et al., 2012; Dezfuli et al., 2018).

It must be stressed that truncal and gastric vagotomies used to be safely performed in human subjects suffering from ulcers, with or without obesity (Gortz et al., 1990; Wills and Grusendorf, 1993). In vagotomized subjects, dumping syndrome, malaise, anemia, and digestive issues commonly occur. However, the latter side effects were usually treatable and not directly correlated with weight loss in many patients (Faxen et al., 1979; Irving et al., 1985; Wills and Grusendorf, 1993). Instead, vagotomy has been reported to induce weight-loss with reduced food intake and modified food preference in obese subjects (Kral, 1978; Gortz et al., 1990). In fact, vagotomized patients often self-reported a lack of hunger (Sheiner et al., 1980; Kral et al., 2009; Plamboeck et al., 2013). The time needed for severed axons to recolonize the human stomach and become functional is unknown. Because peptic ulcers rarely reoccur after vagotomy (Jordan and Thornby, 1987; Popiela et al., 1993), it is unlikely that regrown vagal axons become functional again. Of note, humans treated with truncal vagotomy often received a pyloroplasty to prevent gastric stasis. Pyloroplasty itself exerts profound effects on appetite and food intake that are closely resembling those of bariatric surgery (Fraser et al., 1983; Chang et al., 2001; Dezfuli et al., 2018; Harada et al., 2018). Nonetheless, subsets of patients with selective gastric vagotomy without pyloroplasty also showed long-term weight loss with some degree of dysphagia, early satiety and nausea (Jordan and Thornby, 1987).

### Unintentional Surgical Vagotomy

Certain types of obesity surgeries including, most notably, Roux-en-Y gastric bypass (RYGB) result in a partial and unintentional vagotomy. RYGB is a surgery that consists in dividing the stomach into a small pouch and reconnecting it to the jejunum (Stefater et al., 2012). In bypassed subjects, food travels from the pouch to the lower intestines without traversing the stomach remnant and duodenum (called Roux limb) (Figure 1). Several investigators suggested that RYGB must be accompanied by gastric denervation (Powley et al., 2005b; Berthoud, 2008; Stylopoulos and Aguirre, 2009). Our own experimental data have confirmed that bypassed mice show vagal denervation at the levels of the gastric pouch, the bypassed stomach, and sites of clipping and anastomosis (Gautron et al., 2013). In contrast, vagal innervation remained largely intact in animals that were unoperated on or sham-operated on. In agreement with our observations, RYGB surgery in rats also causes rapid neuronal damage in vagal afferents (Minaya et al., 2019). It is also likely that the construction of a gastric pouch inevitably results in some degree of spinal deafferentation. It is striking that many of the aforementioned effects of RYGB are recapitulated in vagotomized subjects (Brolin et al., 1994; Laurenius et al., 2012; Stano et al., 2017). Moreover, bypassed individuals reach satiation more quickly than expected and independently of side effects such as dumping syndrome and related symptoms such as nausea, cramps, and dizziness (Halmi et al., 1981; Nguyen et al., 2016). Hence, RYGB has a direct impact on the innervation of the stomach, which in turn may actively play a role in the metabolic actions and side effects of gastric bypass. Of note, since food travels directly into the jejunum of bypassed individuals, this new configuration can be conceived as a *functional duodenal vagotomy*, in the sense that duodenal afferents cease to be directly stimulated by nutrients and mechanical stimuli during the postprandial phase (Figure 1). Thus, RYGB is likely to result in a partial loss of sensory input from the upper GI tract including stomach and duodenum. Although counterintuitive, when we consider the literature on intentional and unintentional vagotomies as a whole, it appears that vagotomized animals and human subjects eat less than they did prior to surgery.

### Paradox of Satiation With Vagotomy

It has often been argued, with good reasons, that vagotomies, especially when vagal (motor) efferents are involved, are difficult to interpret because of secondary dysmotility, malaise, and dyspepsia (Sclafani et al., 1981; McDougle et al., 2020). At the same time, the latter factors could not entirely account for reduced food intake and weight loss (Mordes et al., 1979; Sclafani and Kramer, 1985). It is also true that vagotomies are not entirely specific or complete (Browning et al., 2013a) and that experiments consisting of administering exogenous gut peptides, including CCK, lack in physiological relevance (French et al., 1993; Baldwin et al., 1998). Hence, there are numerous unresolved difficulties and discrepancies in the vagotomy literature. Technical and interpretation difficulties aside, one would expect vagotomized individuals to be unable to fully experience satiation and eat more (McDougle et al., 2020). On top of that, the rebound hyperphagia in individuals losing weight when dieting is not seen in bypassed and vagotomized subjects or animals (Berthoud, 2008; Hao et al., 2016). Importantly, bypassed animals are able to overeat upon a metabolic restriction (Stefater et al., 2010; Lutz and Bueter, 2014). This indicates that RYGB does not prevent animals from experiencing hunger but rather produces a state of exacerbated satiation. Thus, subjects with a gastric vagotomy or a truncated stomach likely continue to experience postprandial satiation, if not at exaggerated levels. At first glance, *satiation without a gastric vagus* is a phenomenon that challenges our current understanding of gut-brain communication. RYGB is associated with profound anatomical and physiological changes, the number of which could contribute to perturbate normal eating patterns (Hankir et al., 2018; Sandoval, 2019). For instance, it is possible that RYGB-associated elevated gut peptides secretion contributes to enhanced satiation, among other metabolic improvements. Nonetheless, experts in the field have not reached a consensus on what exactly contributes to altering eating behavior after bariatric surgery (Hao et al., 2016; Sandoval, 2019). For example, RYGB is effective in numerous mouse models lacking key hormones and gut peptides (Mokadem et al., 2014; Morrison et al., 2016; Hao et al., 2018; Boland et al., 2019). Below, I will propose a novel and complementary explanation based on adaptations in viscerosensory circuits.

## The Phantom Satiation Hypothesis

### Overview of Phantom Limbs Phenomena

Is it always the case that the denervation or removal of a body part results in a loss of sensation from this body part? The field of neurology teaches us the contrary. Indeed, many amputees continue to feel the presence of their lost limb, a phenomenon described as *phantom limb* (Ramachandran et al., 2009; Collins et al., 2018; Makin and Flor, 2020). Even though phantom pain syndromes have been first described 500 years ago (Keil, 1990), the treatment of phantom limb syndromes has remained challenging up to this day (Alviar et al., 2016). Briefly, the sensations that can be evoked from a phantom limb can be either non-painful (e.g., tingling), or excruciatingly painful, and spontaneous or consecutive to the stimulation of other body parts (referred sensations) (Aglioti et al., 1994; Ramachandran et al., 2009; Collins et al., 2018). In other words, phantom sensations are sensations felt from a body part that is either missing and/or denervated. The prevalence of phantom pain is estimated at 50–80% of all amputees (Dijkstra et al., 2002). To the best of our knowledge, there is no definitive consensus on how to explain phantom sensations (Ortiz-Catalan, 2018). Among commonly cited reasons for phantom sensations is that damaged sensory fibers in the stump (or neuroma) remain chronically irritated. Their erratic firing may evoke sensations and pain interpreted as originating from the missing body part (Collins et al., 2018). Another explanation involves localized cortical remapping (Flor et al., 2013). The idea behind cortical remapping is that the somatosensory cortical area corresponding to the amputated body part is “invaded” by adjacent cortical areas representing other body parts. Hence, stimuli applied to other body parts may evoke sensations from what is perceived as the missing limb (Hunter et al., 2003). In parallel, maladaptive remodeling of the cortices would contribute to evoking unsolicited pain along with a distorted body representation (Remple et al., 2004). Other researchers have also proposed that large-scale functional changes in brain connectivity beyond the cortex cause phantom sensations (Makin et al., 2013). Lastly, according to a recent theory, amputation renders the neural circuits not normally connected to the missing body part prone to stochastic entanglement with one another (Ortiz-Catalan, 2018). Broadly speaking, the term of entanglement corresponds to the linking of brain networks that do not normally fire together, thereby resulting in unexplained sensations. A combination of all of the above hypotheses, rather than a single mechanism, is likely to account for the emergence of the variety of phantom sensations encountered in amputees.

### Phantom Internal Organs

Understandingly, the literature on phantom sensations focused on limbs, but phantom sensations from internal organs have also been occasionally reported (Roldan and Lesnick, 2014). These organs include the eyes (Andreotti et al., 2014), rectum (Ovesen et al., 1991), kidney (Roldan and Lesnick, 2014), pelvic organs (Dorpat, 1971), and breast (Di Noto et al., 2013; Bjorkman et al., 2017). Of note, Dorpat (1971) already discussed the case of what is called “phantom stomach sensations” but without mentioning satiation itself. In particular, this author mentioned patients complaining of persisting ulcers symptoms after vagotomy or gastrectomy, in spite of their ulcers being healed or removed. Dorpat added that human subjects typically do not experience the feeling of “having an internal organ,” but rather of having sensations normally associated with the functioning of the organ in question. For example, subjects without a rectum sometimes experience the feeling of defecation (Dorpat, 1971; Ovesen et al., 1991). Inspired by the above literature, I would like to propose that satiation continue to arise after gastrectomy or gastric vagotomy due to phantom sensations, which I called the *phantom satiation hypothesis*. Before explaining it, let us note that our hypothesis is speculative and not to be confused with *phantom fullness* that has been used to refer to fullness not correlated with caloric content in healthy individuals (Camps et al., 2016). According to the hypothesis presented here, phantom sensations of satiation are evoked when food is traversing GI segments above and below the denervated or truncated stomach, including the esophagus and lower intestines (Figure 1).

## Predictions and Consistency With the Literature

### Meal Size Reduction

If the hypothesis is correct, phantom satiation should durably influence food intake regulation among bypassed subjects. Accordingly, bypassed individuals report altered experience of hunger and fullness in a qualitative manner (Halmi et al., 1981; Miras and le Roux, 2013). Moreover, bypassed human subjects eat smaller meals for at least two years post-surgery (Laurenius et al., 2012). Overall, reduction in caloric intake over a long period is an important determinant of weight loss after RYGB in humans (Kenler et al., 1990; Odstrcil et al., 2010; Miller et al., 2014). Similarly, meal size reduction was reported in bypassed laboratory animals (Zheng et al., 2009; Mathes et al., 2015; Washington et al., 2016). Several authors postulated that restricting the stomach volume is sufficient to explain a reduced food intake and meal size (Warde-Kamar et al., 2004; Langer et al., 2006). However, others have well explained that gastric capacity is not correlated with reduced feeding after bariatric surgery (Stefater et al., 2012; Sandoval, 2019; Evers et al., 2020). As discussed earlier, gastric vagotomy also causes a reduction in feeding without restricting gastric capacity. Therefore, early satiation in bypassed subjects is highly unlikely to occur because of gastric restriction *per se*, but rather due to changes in the neural circuits normally involved in detecting volumetric changes of the stomach.

### Correlation With Vagal Trauma and Irreversibility

While surgeons pay attention not to damage nerves during bariatric surgery, axons near sites of surgical incision will inevitably be severed. Another prediction is that the degree to which bariatric surgery alters feeding behavior and body weight should be correlated with the trauma to the vagus nerve across different types of bariatric surgeries. For instance, different types of obesity surgeries differently modify the stomach anatomy (Stefater et al., 2012). The two types of surgeries that affect the anatomy of the stomach (and therefore its innervation) the most are RYGB and vertical sleeve gastrectomy (VSG) (Stefater et al., 2010; Kizy et al., 2017). The degree of vagal damage after VSG is poorly defined, but is expected to be extensive along the sites of gastric resection. Interestingly, RYGB and VSG reduce feeding to a comparable extent in humans and rats (Chambers et al., 2011; Stemmer et al., 2013; Yousseif et al., 2014). However, RYGB and VSG exert much more marked effects on feeding behavior than does gastric banding (Korner et al., 2006; Scholtz et al., 2014). One big difference is that gastric banding theoretically does not damage the stomach anatomy or its innervation. Likewise, surgeries associated with incision of gastric tissue produce greater weight loss and reduction of feeding than surgeries only consisting of reducing stomach capacity in the rat (Evers et al., 2020). Following the same idea, because the stomach is more richly innervated than lower GI segments, surgical operation of the lower intestines should affect feeding to a lesser extent. For instance, duodenal-jejunal exclusion, a procedure that leaves the stomach intact, is significantly less effective at reducing feeding or body weight in animals and humans (Geloneze et al., 2009; Kindel et al., 2011; Alvarez et al., 2020). In further support of our model, adding gastric vagotomy to gastrectomy does not produce further weight-loss in laboratory rodents (Hankir et al., 2016; Dezfuli et al., 2018). This is logical considering that gastric vagotomy already occurred in bypassed subjects.

### Irreversibility

Without proper treatment, phantom pain can persist for years in amputees. This may be because the neural changes brought about by an amputation are hardly reversible. RYGB is a complicated procedure that cannot easily be reversed in a laboratory animal. However, reversal of RYGB is performed in patients with severe complications such intractable vomiting, malnutrition or chronic pain (Moon et al., 2015; Shah and Gislason, 2020). As one would expect, reversal of RYGB is often followed by some degree of weight regain consecutive to the correction of the debilitating symptoms (Moon et al., 2015; Shah and Gislason, 2020). When weight regain is observed, most patients do not regain their pre-RYGB weight (Shoar et al., 2016) and, furthermore, a subset of patients never regains any weight. This agrees with the view that the metabolic benefits of RYGB persist to some extent after reversal. Hence, the loss of gastric innervation is a factor that correlate well with the known course of clinical outcomes after bariatric surgery.

### Indiscriminate Weight-Loss in the Non-obese

Our hypothesis implies that a trauma to the upper GI tract will invariably cause weight-loss even in non-obese individuals. Because bariatric surgery is overwhelmingly prescribed for clinically obese subjects, the literature on the impact of bariatric surgery in non-obese is limited. One study in non-obese rats demonstrated significant weight loss after RYGB and VSG (Xu et al., 2015). In contrast, a recent study showed that RYGB did not cause sustained weight loss in the non-obese mouse (Mumphrey et al., 2019). However, RYGB was performed in adolescent mice while they were still growing at a rapid pace. In humans, there is increasing interested in using bariatric surgery to treat diabetic patients without obesity. Several clinical studies have clearly indicated that weight-loss follows RYGB and VSG in non-obese or mildly obese subjects (Cohen et al., 2012; Noun et al., 2016; Ferraz et al., 2019). It is also noteworthy that gastrectomy done in non-obese subjects with gastric cancer also results in sustained weight loss, loss of appetite and abdominal discomfort (Adachi et al., 1999; Laffitte et al., 2015). Notably, surgical reconstruction after various gastrectomies often include Roux-en-Y like procedures, which means that their effects on rate of intestinal nutrient entry and gut hormone secretion will be similar. Hence, gastrectomy and various gastric surgeries indiscriminately causes sustained weight loss.

### Weight Loss and Brain Lesions

In the proposed model, phantom satiation arises following GI denervation and ensuing maladaptive brain changes. One would expect lesions in brain regions involved in processing vagal sensory information including, but not limited to the insula, to be associated with satiation and weight loss. While there is little literature on satiation and brain lesions, neurologists have reported that strokes often cause important weight loss in subsets of patients (Scherbakov et al., 2019). The impact of stroke on body weight differ between patients presumably due to differences in the brain regions affected by the stroke. Taste deficits sometimes manifest alongside post-stroke weight loss (Scherbakov et al., 2011; Dutta et al., 2013), indicating that lesions in viscerosensory brain sites cause weight loss. In one particular clinical case, a patient with a lesion in the left posterior insular cortex, a region that receives general viscerosensory input, showed involuntary weight loss with sustained appetite loss over one year (Mak et al., 2005). We do not dispose of enough evidence to say that exaggerated satiation is responsible for weight loss after brain lesion in humans. Nonetheless, it was repeatedly observed that experimental lesions of viscerosensory relays in non-obese rats including, most notably, the parabrachial and dorsovagal complexes can result in sustained weight loss with depressed ingestive behaviors (Hill and Almli, 1983; Hyde and Miselis, 1983; Kott et al., 1984; Kenney et al., 1989; Dayawansa et al., 2011). In agreement with the hypothesis presented in this article, the aforementioned observations strongly support the idea that derangements in the integrity of key brain sites involved in GI viscerosensory processing can cause weight loss with reduced food intake. In fact, deep brain stimulation for the treatment of human obesity is an active area of research (Nangunoori et al., 2016; Formolo et al., 2019), but is almost entirely focused on brain regions involved in energy balance and reward. Based on the hypothesis presented here, modulating brain regions directly involved in satiation, including the insula and parabrachial complex, may also be considered as an appropriate strategy against obesity.

## Potential Mechanisms Leading to Phantom Satiation

### Nerves Injury and Adaptations

#### Gastric Neuroma

The mechanisms underlying *phantom satiation* are unknown. Nonetheless, I could invoke the same combination of peripheral and central adaptations described in the case of phantom limbs (Figure 1). The neuroma theory postulates that the disorganized growth of damaged nerve terminals at the site of injury can cause phantom sensations including, but not limited to, unexplained pain (Buch et al., 2020). Our group previously reported the presence of dystrophic and damaged vagal terminals in the stomach of the bypassed mouse (Gautron et al., 2013). Dystrophic terminals may correspond to degenerating and/or regrowing axons. Of note, a generalized and persistent decrease in excitability occurs after axotomy of vagal sensory neurons (Scherbakov et al., 2019). Hence, gastric vagal fibers severed during surgery probably become hyporesponsive. As explained before, the vagal supply to the lower GI tract remains largely intact after RYGB (Gautron et al., 2013). In contrast to gastric afferents, the excitability of intact vagal fibers located in the coeliac branches supplying the lower intestines may be enhanced (Figure 1). Specifically, several experimental studies showed that neuronal activation in the nucleus of the solitary tract is greater in RYGB and VSG animals after eating a test meal (Chambers et al., 2012; Mumphrey et al., 2016). Intrajejunal nutrients cause greater satiating effects in bypassed rats (Bachler et al., 2018). The latter findings indicate that vagal afferents that supply the lower intestines behave as if they were hyperresponsive to a meal. What causes the enhanced activity of intestinal vagal fibers is uncertain. Many factors come to mind including rapid gastric emptying, undigested food, excessive gut peptide secretion, altered microbiome, or perturbed vago-vagal reflexes (Sandoval, 2019).

The stomach is also densely innervated by spinal sensory axons traveling through the splanchnic plexus (Spencer et al., 2016). As mentioned before, RYGB surgery is likely to cause spinal endings located in the stomach to be interrupted and damaged. Spinal afferents are critical in visceral nociception (Saper, 2000; Spencer et al., 2016, 2018), but whether irritated spinal endings contribute to a gastric neuroma after bariatric surgery remains to be determined. The hyperexcitability of spinal nociceptors around sites of surgical anastomosis may be one logical explanation of phantom pain, even though no direct evidence is currently available to support this view. Moreover, it is possible that a loss of gastric vagal signaling is indirectly changing the activity of spinal afferent pathways. For example, the electrical stimulation of the vagus nerve produces analgesia by recruiting the antinociceptive descending pathway (Randich and Gebhart, 1992; Janig et al., 2000). Conversely, vagotomy has been associated with hyperalgesia in association with widespread changes in pro-nociceptive pathways stimuli (Ammons et al., 1983; Khasar et al., 1998; Furuta et al., 2009, 2012). Unexplained pain is a key feature of phantom phenomena. In the context of obesity surgery, painful complications with a known etiology can arise (e.g., hernia, adhesion, dumping syndrome) (Iannelli et al., 2006). However, bypassed patients can experience unexplained pain not related to the complications of surgery (Groven et al., 2010; Alsulaimy et al., 2017). For instance, bypassed patients have anecdotally reported feeling general aching all over the body or sharp pain in the stomach area (Groven et al., 2010). At least two studies focusing entirely on the prevalence of post-bariatric chronic abdominal pain revealed that approximately 7% of bypassed patients report pain of unknown etiology (Pierik et al., 2017; Mala and Hogestol, 2018). If unexplained pain is reflective of phantom pain, then the incidence of phantom pain is admittedly much lower after RYGB than after limb amputation. On the other hand, unexplained pain may have been underreported in the literature. According to one study, mild or intermittent pain not requiring a medical follow-up may be present in 80–95% of RYGB patients (Abdeen and le Roux, 2016). Interestingly, persistent abdominal pain occurs in subsets of RYGB patients after reversal to a normal anatomy (Shoar et al., 2016). At the same time, internal organs are less heavily innervated by spinal afferents than limbs and one would expect phantom pain from the upper GI tract to be less severe than in the case of phantom pain from a limb. Finally, it must be noted that the literature on visceral pain after bariatric surgery is still very limited.

#### Dysautonomia

Vagal and spinal afferents are connected in a multisynaptic manner to a complex circuit that exerts a descending excitatory or inhibitory influence on varied autonomic reflexes and sensory pathways (Saper, 2000; Craig, 2002; Travagli et al., 2003; Berthoud et al., 2006). For instance, altered vagal afferents activity may directly modulate vagal efferents pathways in a reflex manner (Travagli et al., 2003). One study also showed that RYGB in the rat leads to both changes in morphology and membrane excitability of vagal (motor) efferents consistent with hyperexcitability (Browning et al., 2013b). Both spinal and vagal afferents also play an important role in modulating the sympathetic outflow to cardiovascular and metabolic viscera (Craig, 2002; Madden et al., 2017; Sabbatini et al., 2017; Rajendran et al., 2019). In particular, the vagus nerve exerts a tonic inhibition on sympathetically-driven thermogenesis in obese rat (Madden and Morrison, 2016). Similarly, vagal afferent signaling is involved in modulating the activity of splanchnic nerves, several of which provide sympathetic innervation to the GI tract, adrenals, and spleen (Sabbatini et al., 2017; Komegae et al., 2018). In other words, gastric vagotomy is likely to cause dysautonomia, a condition in which the parasympathetic and sympathetic outflows to viscera are perturbed (Figure 1). Interestingly, one study recently demonstrated that RYGB significantly elevated the basal activity of sympathetic fibers located in the mouse splanchnic nerve (Ye et al., 2020). The same study also found that splanchnic denervation prevented weight-loss and increase in energy expenditure that normally accompanies RYGB in laboratory animals (Ye et al., 2020). In summary, emerging evidence suggests widespread anatomical and functional changes in sensory and autonomic pathways following RYGB (Figure 1). It must be stressed that cortical and sub-cortical brain regions are linked with autonomic preganglionic and viscerosensory areas by bidirectional neural pathways (Saper et al., 1976; Goto and Swanson, 2004). There is also functional evidence that higher-order brain regions exert top-down excitatory influence on presynaptic vagal terminals (Browning, 2019). Therefore, the aforementioned changes in central circuits and peripheral nerves should be seen as intertwined rather than independent and parallel events (Figure 1). Overall, researchers are just beginning to understand how peripheral neurons are perturbed after bariatric surgery.

### Brain Plasticity and Maladaptive Changes

#### Cortical Remapping

Cortical plasticity has been studied in the context of injuries to somatosensory rather than viscerosensory nerves (Mogilner et al., 1993; Nordmark and Johansson, 2020). Nonetheless, structural and functional changes are likely to occur after bariatric surgery in viscerosensory cortical areas involved in evoking satiation. In particular, after a partial loss of sensory input from the upper GI tract, the area normally representing the denervated region may be taken over by adjacent areas representing nearby innervated GI segments, a phenomenon known as cortical remapping (Figure 1). Whether cortical remapping occurs after bariatric surgery remains unknown. Nerve injury can lead to altered synaptic biology in the mouse insular cortex (Qiu et al., 2014). Surgeries such as mastectomy and hysterectomy are suspected to trigger cortical remapping (Di Noto et al., 2013). Thus, one would expect to see structural and functional changes in the insular cortex and connected integrative cortices after invasive bariatric surgeries including, most notably, RYGB. Nonetheless, studies on cortical remapping in relation to internal organs, in general, and the GI tract, in particular, are very few. If cortical remapping occurs after RYGB, it could explain what I would like to call *referred satiation*. When food is traversing their esophagus and lower intestines in bypassed subjects with truncated and denervated stomach, they may experience a feeling of satiation reminiscent of that normally evoked by gastric distension. This is because the postprandial stimulation of the cortical areas representing the esophagus and lower intestines is interpreted as originating from the stomach after cortical remapping (Figure 1). Perhaps, cortical remapping could explain the exaggerated postprandial neuronal activation and satiation observed in response to jejunum nutrients infusion (Chambers et al., 2012; Mumphrey et al., 2016; Bachler et al., 2018).

#### Brain Dysconnectivity and Neural Entanglement

The altered functional connectivity of long-range subcortical areas involved in viscerosensitivity and nociception may occur (Figure 1). In particular, RYGB has been repeatedly associated with altered functional connectivity in response to food image or food ingestion (Frank et al., 2014; Hunt et al., 2016; Olivo et al., 2017; Baboumian et al., 2019). In the case of food image, brain activity was comparable in normal-weight and RYGB subjects, thus making it difficult to distinguish the contribution of body weight from that of the bypass surgery (Frank et al., 2014). However, in response to food ingestion, brain activity in RYGB patients was significantly different from both normal-weight and obese individuals and weight loss by diet (Hunt et al., 2016; Baboumian et al., 2019). Brain areas differently activated after RYGB encompassed structures distributed across the neuraxis including the hypothalamus, brainstem, hippocampus, and cortex. This suggests that the surgery itself is responsible for the reported changes in brain functions. Many neuroimaging findings in RYGB subjects were often associated with self-reported exaggerated fullness and lower levels of appetite with altered food preferences (Ochner et al., 2011; Hunt et al., 2016; Zoon et al., 2018; Baboumian et al., 2019). Similar brain imaging observations made after VSG (Cerit et al., 2019). Furthermore, widespread structural and functional changes across the brain also take place after RYGB (Rullmann et al., 2018) and VSG (Michaud et al., 2020). Without proving our hypothesis, the above observations are compatible with the idea that large-scale neuroplasticity and maladaptive changes, in both the brain and peripheral nerves, may account for the persistence of satiation and other food-related sensations and emotional responses (Figure 1).

If one applies the stochastic entanglement theory to viscerosensory brain networks (Ortiz-Catalan, 2018), cortical and subcortical regions normally involved in evoking satiation may transition to a state of instability after loss of sensory inputs. When in such a state, neurons belonging to separate viscerosensory modalities may eventually become *entangled* and fire together. The direct result of such a state of viscerosensory entanglement may be postprandial sensations and emotional responses that differ from before surgery. Accordingly, bariatric patients often report new sensations and emotional responses to eating (Hillersdal et al., 2017). Clinicians are also becoming increasingly aware of the risk of new food-related behaviors after bariatric surgery, including anorectic-like behaviors (Opozda et al., 2016; Watson et al., 2020). Because of its stochastic nature (Ortiz-Catalan, 2018), entanglement should be associated with a variability of outcomes after RYGB, both in terms of metabolic benefits (e.g., maximal weigh loss) and side effects (e.g., severity of nausea). In spite of receiving the same surgery, heterogeneity of outcomes among RYGB patients is a well-documented phenomenon (King et al., 2020). This inherent variability in outcomes has complicated the task of determining which exact physiological parameters drive weight-loss in bypassed humans (e.g., feeding vs. energy expenditure). Similarly, in subsets of bypassed subjects, the entanglement of viscerosensory circuits may not occur, potentially leading to failure to lose weight. Certain individuals fail to lose weight after RYGB, often due to excessive eating and psychological issues (Dykstra et al., 2014), with a failure rate estimated at 15–20% (Elnahas et al., 2014; Sima et al., 2019). At first glance, individuals who do not reduce eating after obesity surgery contradict our hypothesis, since the same anatomical changes and degree of trauma to the vagus nerve exist in unresponsive individuals. However, it is noteworthy that phantom sensations, although common in amputees, are not always present. Why certain individuals will not develop phantom sensations, or only temporarily, remains unclear (Ortiz-Catalan, 2018). Perhaps, failure to lose weight after bariatric surgery is related to genetic polymorphisms affecting the neural circuits involved in appetite regulation. A good example is the melanocortin-4-receptor (MC4R), a brain and vagal receptor involved in appetite regulation (Cone, 2006; Gautron et al., 2010). Impaired MC4R signaling in both humans and animals is associated with resistance to RYGB-induced weight loss (Hatoum et al., 2012; Zechner et al., 2013). Gene variations involved in neurotransmitters and gut peptides signaling have also been linked to the outcome of RYGB (Matzko et al., 2012; Novais et al., 2016). This further underscores the critical role that the nervous system plays in the metabolic outcome of obesity surgeries. Alternatively, the entanglement theory may also account for the occasional failure of RYGB. While it remains a speculative theory, the idea of entanglement of viscerosensory functions fits remarkably well with a large body of clinical observations pertinent to bypassed subjects.

## Phantom Satiation and Weight-Loss

### Does Satiation Account for Weight-Loss After RYGB?

One question to ask is whether phantom satiation can contribute to sustained weight loss. It has been argued that reduced meal size in bypassed laboratory animals is unlikely to drive weight loss because, unlike in humans, they tend to consume the same number of daily calories as experimental controls over the long term (Zechner et al., 2013; Mokadem et al., 2014; Arble et al., 2018). In fact, bypassed animals progressively eat more meals in a compensatory manner (Zheng et al., 2009). Instead, a combination of elevated energy expenditure and malabsorption contribute to weight loss in animal models of bariatric surgery (Bueter et al., 2010; Li et al., 2015; Ye et al., 2020). Many biological and technical factors may possibly account for species differences in the outcome of bariatric surgery (Sandoval, 2019). In particular, the anatomical organization of viscerosensory circuits may differ between primates and rodents (Craig, 2002). Moreover, metabolic regulation differs in significant ways between humans and laboratory rodents (Even et al., 2017). Here, I suggest that phantom satiation can partially contribute to weight loss, even in the case of unchanged total daily food intake. Indeed, emerging evidence points to the fact that meal size and feeding timing contribute to the etiology of human obesity, independent of changes in total food intake (Berg et al., 2009; Mattson et al., 2014; Baron et al., 2017; McHill et al., 2017). Moreover, inherent circadian variations in central and peripheral metabolic pathways exist (Hatori et al., 2012; Cedernaes et al., 2019). Remarkably, constraining laboratory rodents to eating small intermittent meals is sufficient to prevent hyperphagia and diet-induced obesity even in association with unchanged daily caloric intake (Licholai et al., 2018). Hence, it is plausible that phantom satiation, by constraining meal size and patterns throughout the day, may contribute to weight loss (Figure 1). Our hypothesis is focused on satiation and feeding behavior but, by no means, excludes the contribution of altered energy expenditure to the health benefits of bariatric surgery. In fact, food intake and energy expenditure are more intertwined than often considered. For example, the simple act of sham feeding (chewing followed by spitting) can raise energy expenditure in human subjects (LeBlanc and Cabanac, 1989; LeBlanc and Soucy, 1996). The latter phenomenon has been described as a *cephalic thermic response to food*. Thus, it may be that eating smaller meals more often throughout the day also augments thermogenesis and energy expenditure. That said, gastric denervation cannot account for the entirety of the metabolic effects of bariatric surgery because gastric vagotomy alone is less effective than RYGB. However, this could also be because vagotomy alone does not entirely recapitulate the impact of RYGB on the non-vagal components of the stomach innervation. Lastly, the hypothesis described in this article deliberately focused on satiation because it is a well-known function of vagal signaling in the upper GI tract (Bai et al., 2019). Nonetheless, the vagus nerve is involved in modulating food-related sensations and feelings other than satiation including, but not limited to, food reward, appetition, learned taste avoidance, food preference, and nausea (Sclafani and Kramer, 1985; Labouesse et al., 2012; Sclafani and Ackroff, 2012; Horn, 2014; Behary and Miras, 2015; Hankir et al., 2017; Han et al., 2018; Shechter and Schwartz, 2018; Qu et al., 2019; Sandoval, 2019; Fernandes et al., 2020; Zhang et al., 2020). It is therefore conceivable that phantom sensations arising after bariatric surgery may include phantom nausea and discomfort, as well as taste disturbance and modified food preferences. In apparent agreement with this view, food preference after bariatric surgery shifts away from caloric and fatty food (Chambers et al., 2012; Nielsen et al., 2019). Furthermore, using direct measurements of food intake, human studies showed that weight loss is correlated with a marked reduced preference for caloric food after bariatric surgery, but only in certain individuals (Sondergaard Nielsen et al., 2018). However, food preferences are not significantly changed by surgery in most patients (Sondergaard Nielsen et al., 2018) and, consequently, are unlikely to play a major role in the metabolic outcomes of either RYGB or VSG in humans.

As a side note, RYGB and VSG rapidly improves glucose homeostasis in both human subjects and laboratory animals (Bojsen-Moller, 2015; Arble et al., 2018). At first glance, the hypothesis described in this article does not seem relevant to glucose metabolism because the anti-diabetic actions of these surgeries occur independently of weight-loss and reduced feeding (Chambers et al., 2011; Laferrere and Pattou, 2018; Evers et al., 2020). On the other hand, eating smaller meals may contribute to improve glucose metabolism after RYGB in human subjects (Stano et al., 2017). Moreover, peripheral nerves are known to play a modulatory role in glucose metabolism (Gardemann et al., 1992; Cardin et al., 2001; Razavi et al., 2006). For instance, gastric vagotomy alone alters glucose homeostasis in humans (Akiyama et al., 1984; Galewski et al., 1995; Plamboeck et al., 2013) and, furthermore, an intact innervation to the portal vein is required for the anti-diabetic actions of RYGB in mice (Troy et al., 2008). Therefore, the hypothesis described in this article may also be relevant to glucose metabolism after bariatric surgery.

### Mimicking Phantom Satiation: A New Weight-Loss Treatment?

The second question to ask is whether the phantom satiation hypothesis could help design a new weight-loss treatment. I concur with the view that a better understanding of the biological changes taking place after bariatric surgery may lead to the discovery of a novel and less invasive weight loss treatment (Browning and Hajnal, 2014; Pucci and Batterham, 2019; Gimeno et al., 2020). If the *phantom satiation* hypothesis is correct, the biological changes that accompany bariatric surgeries are highly complex and widespread across the body all the way up to integrative cortices (Figure 1). This level of complexity may render difficult finding alternative strategy to bariatric surgery, especially by pharmacological means. With this in mind, a successful alternative to bariatric surgery may consist in permanently silencing the nerves to the upper GI tract, by device- or surgery-assisted means. In support of this view, obese patients are already eligible for an FDA-approved vagal neuromodulation procedure termed vBloc (Shikora et al., 2015; Apovian et al., 2017). Neuromodulatory devices can be laparoscopically implanted and electrodes attached to each vagal trunk near the gastro-oesophaeal junction. The stimulation at the high frequency of at least 500 Hz inhibits the axonal conduction of both motor and sensory neurons (Camilleri et al., 2008; Waataja et al., 2011). Its mechanisms of action remain mysterious and one recent modeling study indicates that vBloc is more likely to excite than inhibit vagal afferents (Pelot et al., 2017). On the other hand, new data in rats also indicate that electrodes chronically attached to the vagus nerve produce significant damage to vagal efferent axons (Somann et al., 2018). Patients can expect an average excess weight loss of 22% over 18 months, which is admittedly not as effective as RYGB. However, vBloc differs in significant ways from bariatric surgery. First, vBloc only stimulates the vagus nerve in an intermittent manner and, secondly, leaves spinal nerves untouched. Regardless of technical details, the *phantom satiation* hypothesis predicts that an effective alternative strategy to bariatric surgery would consist in mimicking, as closely as possible, the patterns of denervation and dysautonomia observed after RYGB. In the anticipation of side effects, such an alternative strategy should ideally be easily reversible and adjustable in strength.

## Concluding Remarks

To paraphrase Dr. David Horrobin, a hypothesis that is wrong will attract no following and disappear (Horrobin, 2000). If proven right, however, it can be the beginning of new fields of knowledge. As of today, the *phantom satiation* hypothesis appears consistent with a large body of literature and provides a complementary explanation to already existing hypotheses on how GI surgeries modify metabolism and behaviors. Many researchers have stressed the key role played by the brain and peripheral nerves in the outcome of bariatric surgery (Powley et al., 2005b; Browning and Hajnal, 2014; Lasselin et al., 2014; Li and Richard, 2017; Hankir et al., 2018; Sinclair et al., 2018; Sandoval, 2019; Nota et al., 2020; Ye et al., 2020). Specifically, they have proposed that microbial, hormonal, inflammatory and autonomic changes may contribute, perhaps synergistically, to alter brain functions after bariatric surgery. In turn, altered brain activity may contribute to modified energy expenditure with or without altered food intake. Together, the *phantom satiation* hypothesis and related neurocentric hypotheses of bariatric surgery invite us to consider the GI tract not merely as a receptacle for food but as a complex sensory organ. Nonetheless, the present hypothesis differs from other theories that convey the idea that bariatric surgery “corrects” biological derangements brought about by obesity. Yes, bariatric surgery is genuinely effective, but it should not be touted as a “corrective procedure,” considering that its mechanisms remain uncertain. In particular, available data suggest that vagal and GI functions remain largely normal in obesity (Lieverse et al., 1994; Klatt et al., 1997; Bluemel et al., 2017). In other words, bariatric surgery is done on an otherwise healthy GI tract, which inevitably deteriorates, rather than improves, its functions. The best evidence is that gut peptide secretion in bypassed subjects is very high compared to that of normal-weight subjects (Laferrere et al., 2007; Dirksen et al., 2013). Furthermore, the intestinal mucosa of bypassed human subjects and animals becomes abnormally hypertrophic (le Roux et al., 2010; Mumphrey et al., 2015). Hence, if the *phantom satiation* hypothesis is correct, bariatric surgery is not a procedure that returns obese individuals to some hypothetical state of “normalcy” but rather that profoundly disrupts normal GI viscerosensory and endocrine functions (Figure 1). To be clear, the phantom satiation hypothesis should not be interpreted as if humoral mechanisms are irrelevant to the outcome of bariatric surgery. In particular, the receptors for many gut peptides are expressed in brain regions involved in viscerosensory functions (Honda et al., 1993; Zigman et al., 2006; Graham et al., 2020). It is therefore conceivable that excessive gut peptides secretion after bariatric surgery may contribute to exacerbate the aforementioned brain maladaptive changes observed after vagal damage. Hence, further understanding of the mechanisms underlying bariatric surgery will likely require a multidisciplinary and integrated approach beyond the traditional boundaries of the academic disciplines of endocrinology and metabolism. In particular, one area in need of further research is how bariatric surgeries perturbate interoceptive, nociceptive, and autonomic pathways.

## Data Availability Statement

The original contributions presented in the study are included in the article/supplementary material, further inquiries can be directed to the corresponding author.

## Author Contributions

LG wrote the manuscript entirely and generated the figure.

## Conflict of Interest

The author declares that the research was conducted in the absence of any commercial or financial relationships that could be construed as a potential conflict of interest.
